# Nephroprotective Effects of *Tanacetum balsamita* Extract on Metabolic-Induced Renal Injury (MIRI) in Rats

**DOI:** 10.3390/cimb47040293

**Published:** 2025-04-21

**Authors:** Rumyana Simeonova, Reneta Gevrenova, Lyubomir Marinov, Yonko Savov, Dimitrina Zheleva-Dimitrova

**Affiliations:** 1 Department of Pharmacology, Pharmacotherapy and Toxicology, Faculty of Pharmacy, Medical University of Sofia, 1000 Sofia, Bulgaria; lmarinov@pharmfac.mu-sofia.bg; 2 Department of Pharmacognosy, Faculty of Pharmacy, Medical University of Sofia, 1000 Sofia, Bulgaria; rgevrenova@pharmfac.mu-sofia.bg; 3 Institute of Emergency Medicine “N. I. Pirogov”, Bul. Totleben 21, 1000 Sofia, Bulgaria; yonko_savov@hotmail.com

**Keywords:** *Tanacetum balsamita*, diabetes type 2, metabolic syndrome, diabetic nephropathies, oxidative stress

## Abstract

The progression of type 2 diabetes is associated with multiple complications, one of which is diabetic nephropathy (DN). This study aimed at investigating the nephroprotective potential of two doses 150 mg/kg and 300 mg/kg of *Tanacetum balsamita* leaf extract (ETB) on metabolic-induced renal injury (MIRI) in rats. Markers of renal oxidative stress and antioxidant defense, histopathology, serum biochemistry, and urinalysis were measured. Blood glucose level and arterial blood pressure were assessed weekly for the experimental period of eight weeks. ETB at a high dose significantly decreased the blood glucose levels and mildly lowered systolic pressure in diabetic rats. In the kidney, ETB restored the antioxidant marker malondialdehyde, reduced glutathione, and markedly increased enzymatic activity related to GSH turnover by 46% (GPx), 22% (GR), 32% (GST), and 96% (SOD). ETB reduced elevated urea and creatinine levels and alleviated the proteinuria along with other urinalysis parameters. Histopathological examination of the kidney supported the observed protective effects. Both doses of the ETB ameliorated most of the investigated parameters similarly to positive controls enalapril and acarbose. ETB benefits on MIRI-induced damages could be associated with high levels of mono- and dicaffeoylquinic acids together with a series of methoxylated flavones and flavonols, which may hold significance for its antidiabetic and nephroprotective activity.

## 1. Introduction

Diabetes is associated with an increased risk of heart disease, stroke, high blood pressure, atherosclerosis, liver abnormalities, and others. Diabetes is also the main cause of kidney disease. About one in three adults with diabetes has kidney disease or diabetic nephropathy (DN) [1]. Despite major advances in the understanding of the pathogenesis of DN and approaches to slow its development and progression, effective therapeutic modalities for its treatment are currently unavailable. Treatment is difficult and expensive.

The main and most important groups of drugs that can improve the condition of patients with MIRI are renin-angiotensin-aldosterone system (RAAS) inhibitors, sodium-glucose cotransporter-2 (SGLT2) inhibitors, glucagon-like peptide-1 (GLP-1) agonists, and nonsteroidal mineralocorticoid receptor antagonists (nsMRAs) [2].

Even with therapeutic advances, DN remains the principal cause of end-stage renal disease (ESRD), highlighting the need for continued research to identify new biomarkers and innovative treatments [3]. The use of multiple synthetic drugs reduces the patient’s compliance and leads to various adverse effects that ultimately worsen the patient’s condition. Therefore, alternative strategies to prevent or treat DN are urgently needed. Bioactive compounds in herbal products that can improve the condition of patients with diabetes and its complications are safer and, in some cases, effective enough.

The scientific literature abounds with examples of antioxidant, antidiabetic, and nephroprotective effects of extracts and isolated bioactive substances of plant origin [4,5]. Hence, therapies targeting oxidative stress and inflammation may effectively preserve normal renal function and delay the progression of MIRI. In our previous investigation on the antidiabetic potential of extract from *Tanacetum balsamita* leaves (ETB), we displayed the potential of this plant species to alleviate type 2 diabetes symptoms and metabolic-associated fatty liver disease (MAFLD) in experimental models [6]. At 300 mg/kg, ETB significantly reduced the blood glucose levels; decreased lipase activity, total cholesterol, and triglycerides; and restored the amylase activity compared to the that in the MAFLD group. The beneficial effects of ETB were manifested by increasing the activity of antioxidant enzymes, and ameliorating oxidative stress biomarkers reduced glutathione and malondialdehyde in comparison with the MAFLD rats. Importantly, ETB prevents the histopathological changes related to MAFLD.

Although *Tanacetum* species are drawing increased interest from medical and nutritional scientists, most of the research has been focused on the benefits of essential oils and sesquiterpene lactones [7,8].

A previous study on the *T. balsamita* leaves, roots, and flower heads allowed for the dereplication/annotation of more than 100 specialized metabolites by means of ultra-high-performance liquid chromatography–high-resolution mass spectrometry [9]. The methanol-aqueous extract from leaves exerted antioxidant activity in radical scavenging, reducing the power and metal chelating assays. The high antioxidant potential was related to the phenolic and flavonoid content reaching 30.82 mg gallic acid equivalent (GAE)/g dry extract (de) and 18.97 mg quercetin equivalent (QE)/g de, respectively. The polyphenolic profiling described in the aforementioned study was delineated by acylquinnic acids (chlorogenic, neochlorogenic, 3,4-dicaffeoylquinic, and 3,5-dicaffeoylquinic acid), coumaric acid-*O*-hexoside, hexuronide of luteolin, chrysoeriol, and jaceosidin together with the aglycones nepetin and jaceosidin. A series of caffeic acid conjugates was determined by ultra-high-performance liquid chromatography with diode array detection (UHPLC-DAD) [6]. The main compound was 3,5-dicaffeoylquinic acid, being present at 69.13 μg/mg de, followed by chlorogenic acid (17.97 μg/mg de) and rosmarinic acid (12.95 μg/mg de). In addition, the dominant flavonoids were isorhamnetin 3-*O*-glucoside (5.72 μg/mg de), orientin (4.60 μg/mg de), and hyperoside (3.92 μg/mg de). It is noteworthy that the unique nature of the health-promoting benefits of *T. balsamita* leaves, including increased antioxidant production, has been associated with naturally high levels of acylquinic acids [6,9]. Among them, chlorogenic acid (CGA) and 3,5-DCQA deserve special attention. Both compounds have been found in abundance in numerous Asteraceae taxa [10,11,12,13]. They exert diverse therapeutic effects in chronic metabolic diseases and age-related disorders. Experimental models in rodents have traditionally been used to study the pathogenesis of diabetes, its complications, and the development of new therapeutic strategies. Despite the accumulating evidence that caffeoylquinic acids and leaf extracts from *Tanacetum* species protect against oxidative stress during liver and brain injury in vivo, the nephroprotective effects have not been thoroughly investigated. Our objective was to examine whether *T. balsamita* leaf extract ameliorates MIRI created by the administration of a high-calorie diet (high-fat diet (HFD)) and a single i.p. administration of nicotinamide and streptozotocin (STZ) [14]. The main compound was 3,5-dicaffeoylquinic acid, being present at 69.13 μg/mg de, followed by chlorogenic acid 17.97 μg/mg de and rosmarinic acid 12.95 μg/mg de. Isorhamnetin 3-*O*-glucoside, orientin, and hyperoside levels were 5.72, 4.60, and 3.92 μg/mg de.

## 2. Materials and Methods

### 2.1. Animals

Forty male Wistar rats with body weights of 150–180 g were kept in Plexiglas cages (5 per cage) in a 12/12 light/dark cycle, under standard laboratory conditions (ambient temperature 20 ± 2 °C and humidity 72 ± 4%). All animals were purchased from the National Breeding Center, Sofia, Bulgaria and allowed a minimum of 7 days to acclimatize before the start of the study. Food and fresh drinking water were provided ad libitum. All procedures involving animals were approved by the Animal Care Ethics Committee from the Bulgarian Food Safety. An ethical clearance (No. 346 of 28.02.2023) was issued.

### 2.2. Plant Material, Sample Extraction, and Phytochemical Analyses

Plant material (leaves of *T. balsamita*) were collected from herbal garden (Belopoptsi village, Gorna Malina region) in Bulgaria at 700 m a.s.l. (42.67° N, 23.77° E) during the full flowering stage in July 2022. Voucher specimen was deposited at Herbarium Academiae Scientiarum Bulgariae (SOM 177 806). Air-dried powdered leaves (50 g) were extracted with 80% MeOH (1:20 *w*/*v*) by sonication (100 kHz, ultra-sound bath Biobase UC-20C, Jinan, Shandong, China) for 15 min (×2) at room temperature. The extract was concentrated in vacuo, defatted with CH_2_Cl_2_, and lyophilized (lyophilizer Biobase BK-FD10P, Jinan, Shandong, China) to yield crude lyophilized extract (ETB) used for further in vivo analyses [6]. ETB was analyzed by ultra-high-performance liquid chromatography–high-resolution mass spectrometry (UHPLC-HRMS) and ultra-high-performance liquid chromatography–Diode Array Detection (UHPLC-DAD), as previously described [6,9].

### 2.3. Experimental Design

This study aimed to investigate the effects of ETB, administered orally at two doses (150 mg/kg and 300 mg/kg [6]) to male normal rats and rats with metabolic-induced renal injury (MIRI). In order for them to develop MIRI, rats were fed with a high-fat diet (HFD) and additionally received 10% fructose for drinking [15]. On the 21st day, they were challenged with 45 mg/kg, i.p. streptozotocin (STZ) dissolved in citrate buffer 0.1 M, pH 4.4, 15 min after the i.p. administration of nicotinamide (NA, 110 mg/kg bw) [16]. Rats with blood glucose concentration over 8 mmol/L were regarded as successfully established models of MIRI.

The effects of ETB were compared with those of the positive controls, enalapril and acarbose. MIRI is associated with hyperglycemia and oxidative stress which activate the RAAS system and in turn lead to an increase in blood and intraglomerular pressure as well as additional kidney damage [17]. Enalapril is widely used in clinical practice, not only to reduce high blood pressure, but also to slow the progression of kidney damage in patients with diabetic nephropathy. Acarbose was used as a second positive control because of the previously found good alpha-glucosidase inhibitory potential of *T. balsamita* [6,9].

Forty male Wistar rats were randomly divided into eight groups of five animals (n = 5) as follows:

Group 1—control group animals, with free access to fresh water and normal pelleted food;
Group 2—rats orally treated with low dose (150 mg/kg/p.o.) of extract of *T. balsamita* (ETBld) for 8 weeks;
Group 3—rats given high dose (300 mg/kg/p.o.) of extract of *T. balsamita* (ETBhd) for 8 weeks;
Group 4—rats with induced metabolic syndrome and subsequent MIRI;
Group 5—MIRI group treated orally once a day with enalapril (enlp, 5 mg/kg/day) [18] (from 5th to 8th week) as the positive control for hypertension;
Group 6—MIRI rats treated orally once a day with acarbose (5 mg/kg) [19] (from 5th to 8th week) as the positive control for diabetes type 2 (DT2);
Group 7—MIRI rats treated with ETBld for the whole period (from 1st week to the end of the 8th week);
Group 8—MIRI rats treated with ETBhd for the whole period;

During the entire eight-week experimental period, Groups 4, 5, 6, 7, and 8 received an additional 10% fructose (HF) solution for drinking.

Blood glucose level and arterial blood pressure of the experimental animals were measured once a week for 8 weeks. Measurement of arterial blood pressure was performed using a CODA^®^ non-invasive blood pressure system from Kent Scientific, Torrington, Connecticut, CT 06790, USA that has been described previously [15]. In the last, eighth week, rats from all experimental groups were placed in metabolic cages for 24 h and urine was collected for test strip testing.

At the end of the experimental period at the 57th day after overnight starvation, the animals were euthanized, blood was collected, and the levels of urea and creatinine in the serum were measured. Afterwards, the kidneys were taken and immediately transferred to ice-cold containers, weighed, and homogenized to assess the oxidative stress biomarkers malonedialdehyde (MDA) and reduced glutathione (GSH) as well as the activity of the antioxidant enzymes glutathione peroxidase (GPx), glutathione reductase (GR), glutathione-S-transferase (GST), and superoxide dismutase (SOD). Small pieces from the kidneys were taken and fixed in 10% buffered formalin for histopathological investigation.

### 2.4. Assessment of Serum Biochemical Parameters

The weekly measurement of blood glucose levels was performed by test strips for mini-system MULTICAREIN (BSI Diagnostics, Arezzo, Italy) using a blood drop from the tail vein. At the end of the experimental period (8 weeks), the biochemical serum data characterizing kidney function, namely, urea and creatinine, were measured using automated biochemistry analyzer kits (BS-120, Mindray, Shenzhen, China), following the manufacturer’s instructions.

### 2.5. Urinalysis

The urine volume, collected for 24 h; protein and glucose presence in the urine; pH; and ketones were assessed using urine reagent strips for urinalysis “Condor-Teco Medical Technology-China”.

### 2.6. Assessment of the Oxidative Stress Biomarkers

Oxidative damage was determined in kidney homogenates by measuring spectrophotometrically at 535 nm the quantity of thiobarbituric acid-reactive substances (TBARS), expressed as malondialdehyde (MDA) equivalents as described by Polizio and Peña [20]. The concentration of MDA was calculated using a molar extinction coefficient of 1.56 mM^−1^ cm^−1^ and expressed in nmol/g wet tissue. Reduced glutathione (GSH) was assessed in kidney homogenates by measuring the non-protein sulfhydryls after precipitation of proteins with trichloroacetic acid (TCA), using the method described by Bump [21]. The absorbance was determined at 415 nm, and the results were expressed as nmol/g wet tissue. The antioxidant enzymes activities were measured in the supernatant of 10% kidney homogenates, prepared in 0.05 M phosphate buffer (pH 7.4). The protein content of kidney homogenate was measured spectrophotometrically at 750 nm using the method of Lowry [22]. Glutathione peroxidase activity (GPx) was measured spectrophotometrically at 340 nm using a coupled reaction system consisting of GSH and glutathione reductase and an extinction coefficient of 6.22 mM^−1^ cm^−1^ [23]. Results are expressed in nmol/mg protein/min. Glutathione reductase activity (GR) was measured spectrophotometrically according to the method of Pinto et al. [24] at 340 nm and using an extinction coefficient of 6.22 mM^−1^ cm^−1^. Glutathione-S-transferase activity (GST) was measured spectrophotometrically at 340 nm using 1-chloro-2,4-dinitrobenzene (CDNB) as a substrate [25]. The enzyme activity is expressed as nmol of CDNB-GSH conjugate formed/min/mg protein. Superoxide dismutase activity (SOD) was measured according to the method of Misra and Fridovich [26] following the autoxidation of epinephrine and using the molar extinction coefficient of 4.02 mM^−1^ cm^−1^.

### 2.7. Histopathological Examination

Histopathological examination of the kidneys was performed using the method of Bancroft and Gamble [27]. For light microscope evaluation, kidney tissues were fixed in 10% buffered formalin, and thin sections (4 μm) were subsequently stained with hematoxylin/eosin for general histological feature determination. The sections were observed under high-power microscope, and photomicrographs were taken using “Olympus” CX31 (Karl Zeiss, Oberkochen, Germany) and Camera “Olympus x Optical zoom” with objective “PlanaC” 4/0.10 (Karl Zeiss, Oberkochen, Germany).

### 2.8. Statistical Analysis

The statistical program ‘MEDCALC’ version 23.2.1 was used for the analysis of the in vivo data. Results are expressed as mean ± SD for five animals in each group. Comparisons within two groups were made by Student′s *t*-test. One-way analysis of variance (ANOVA) with post hoc multiple group comparisons (Dunnet *t*-test) was used to assess statistical differences. Values of *p* < 0.05 were considered statistically significant.

## 3. Results

UHPLC-HRMS and UHPLC-DAD analyses for the qualitative and quantitative determination of the secondary metabolites in ETB have been previously described [6,9]. Chromatograms and the detailed composition of the extract are presented in Figures S1 and S2 and Tables S1 and S2. The main compounds in the tested extract were the phenolic acids 3,5-dicaffeoylquinic acid, chlorogenic acid, and rosmarinic acid, as well as the flavonoids isorhamnetin 3-*O*-glucoside, orientin, and hyperoside (Figure S2, Table S2).

### 3.1. Assessment of Blood Glucose Level and Systolic Blood Pressure

**Figure 1:**
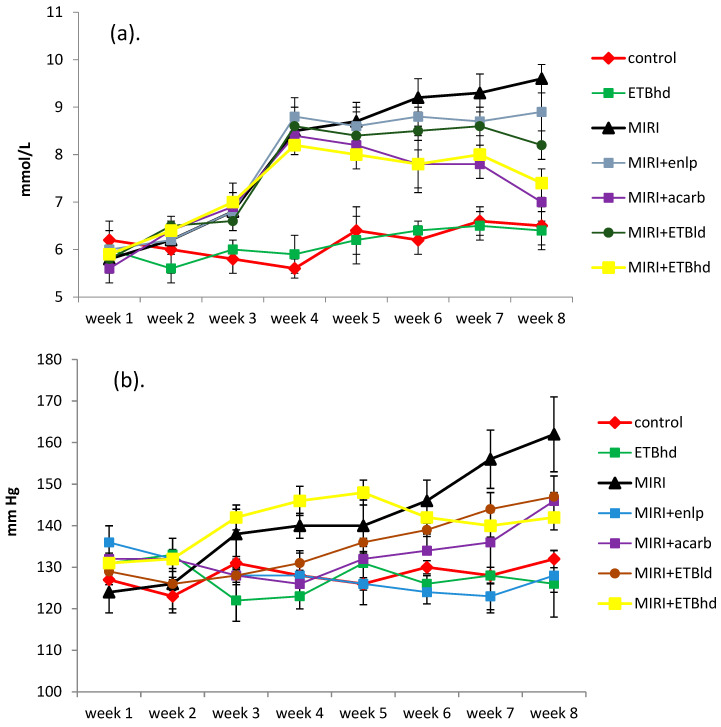
Weekly blood glucose level changes in mmol/L (**a**); weekly systolic blood pressure changes in mm Hg (**b**). Results are expressed as mean ± SD (n = 5). *Abbreviations*: ETBld, extract of *T. balsamita*, low dose (150 mg/kg); ETBhd, extract of *T. balsamita*, high dose (300 mg/kg); MIRI, metabolic-induced renal injury; MIRI + enlp, metabolic-induced renal injury + enalapril; MIRI + acarb, metabolic-induced renal injury + acarbose; MIRI + ETBld, metabolic-induced renal injury + extract of *T. balsamita*, low dose (150 mg/kg); MIRI + ETBhd, metabolic-induced renal injury + extract of *T. balsamita*, high dose (300 mg/kg).

In the present study, we found that after the induction of diabetes on day 21, there was a sharp rise in the blood glucose level in all experimental groups. These values remained relatively high until the end of the experiment in the model pathology group and the enalapril-treated metabolic animals, whose blood sugar was, respectively, 48% and 40% higher than the control blood sugar on that day. On day 57, there were statistically significant reductions of 27% and 23% in blood sugar levels in the acarbose and high-dose extract-treated diabetic animals, respectively, compared to the untreated diabetic animals (Figure 1a).

Diabetes and hypertension often coexist and form the most unfavorable combination, diabetic nephropathy. By affecting high blood glucose and hypertension, kidney function could be significantly improved. At the end of the experimental period, a statistically significant increase of 23% was recorded in systolic blood pressure in the MIRI-induced animals compared to the control group on that day (Figure 1b). A 21% reduction in systolic blood pressure at day 57 was only observed in the diabetic animals treated with enalapril, which is not surprising since enalapril is an example of an antihypertensive drug. Metabolic rats treated with a high dose of the extract also showed a slight decrease in blood pressure of 13% compared to that of the untreated group with induced pathology. Acarbose and low-dose extract did not statistically significantly affect arterial blood pressure (Figure 1b).

### 3.2. Oxidative Stress Biomarkers

**Figure 2:**
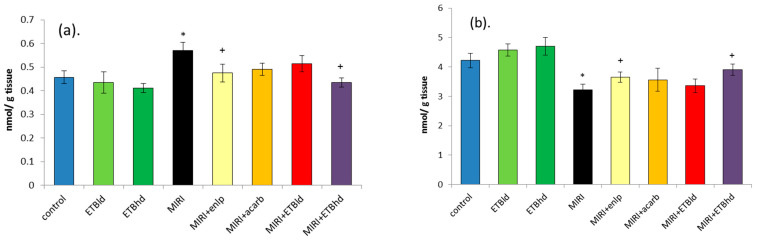
Levels of malondialdehyde (MDA) (**a**) and reduced glutathione (GSH) (**b**) in the kidney homogenates. Results are expressed as mean ± SD (n = 5). * *p* < 0.05 vs. control; + *p* < 0.05 vs. MIRI; treatment: as described in the experimental design section.

Oxidative stress is a significant factor in kidney disease, marked by an imbalance between the generation of ROS and the body’s antioxidant mechanisms. Hyperglycemia, a prevalent characteristic of diabetes and DN, is a significant catalyst for oxidative stress [28]. The obtained results reveal the changes in the levels of MDA (a) and GSH (b) in the kidneys of the groups of animals studied (Figure 2). In the kidneys of rats with MIRI, the amount of MDA was statistically significantly higher by 24%, and the level of GSH was 24% lower than that in the corresponding control group.

In the present study, we also confirmed that this pathology was associated with a significant decrease in the activity of antioxidant enzymes GPx and GST by about 31%, GR activity by 21%, and SOD activity by 28% compared with that in the control animals (Table 1).

The administration of enalapril and the high dose of extract to the animals with MIRI significantly reduced the increased level of MDA by 17% and 24%, respectively, compared to that in untreated rats with induced METS. The renal GSH level increased by 13% and by 21% in enalapril- and high-dose-extract-treated animals, respectively. These changes suggest an antioxidant effect of both enalapril and the extract, which is confirmed by the measured activity of antioxidant enzymes. Enalapril significantly increased the activity of GPx by 35%, of GR by 15%, of GST by 38%, and SOD by 78% relative to those in the untreated MIRI animals (Table 1).

In the present experiment, administration of both doses of extract resulted in an increase in the activity of GPx by 30% and by 46%, of GR by 17% and by 22%, of GST by 24% and by 32%, and of SOD by 91% and 96%, respectively, relative to the those in the kidneys of untreated animals with MIRI (Table 1).

### 3.3. Assessment of Serum Biochemical Parameters and Urinalysis

**Figure 3:**
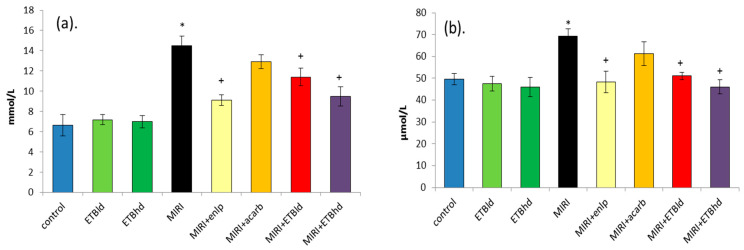
Concentrations of urea (**a**), and creatinine (**b**) in the serum of the experimental groups. * *p* < 0.05 vs. control; + *p* < 0.05 vs. MIRI; treatment: as described in the experimental design section.

MIRI is confirmed by measuring blood urea and creatinine levels as well as proteinuria. Among the biochemical markers characterizing renal function, these markers were measured in the present study (Figure 3, Table 2).

Both serum indicators had higher values in diabetic animals with induced MIRI, urea by 118% and creatinine by 40%, respectively, compared to those in the control healthy rats. Enalapril reduced the increased values of urea and creatinine by 37% and 30%, respectively, compared to those in diabetic animals.

### 3.4. Histopathological Findings

**Figure 4:**
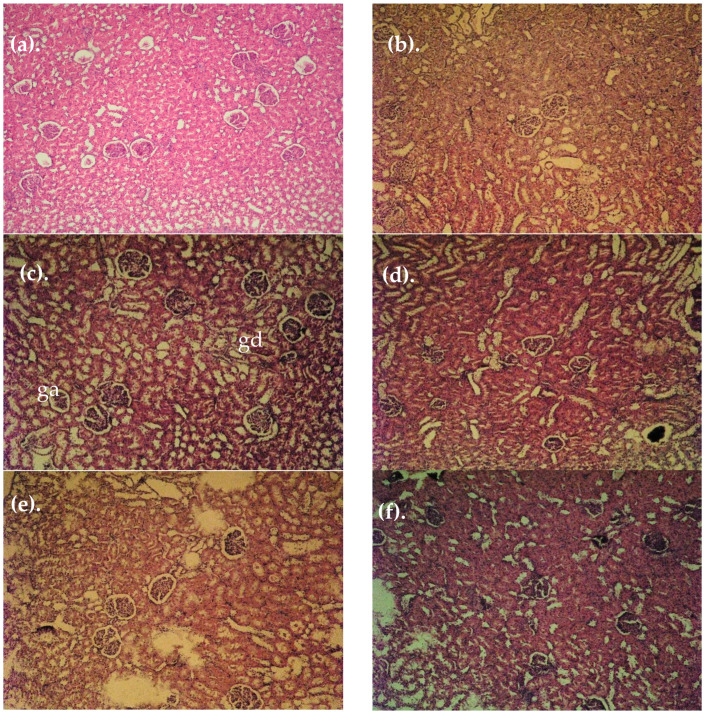
Histopathological changes in the kidneys of animals from experimental groups: (**a**). Controls; (**b**). ETBhd; (**c**). MIRI; (**d**). MIRI + enlp; (**e**). MIRI + acarbose; (**f**). MIRI + ETBhd; glomerular atrophy (ga) and granular degeneration (gd).

Histopathological changes in the architecture of the renal tissue confirm the already established biochemical disorders in animals with MIRI (Figure 4). In the kidneys from control animals and in animals treated with extract alone, no visible changes in morphology in the tubular and glomerular apparatus were observed (Figure 4a,b). In the MIRI group, diabetically altered kidneys with diffuse interstitial tubular and glomerular damage with glomerular atrophy (ga) and granular degeneration (gd) were seen. As a consequence of diabetes and hypertension, a diabetically altered kidney with diabetic intracapillary glomerulosclerosis was observed (Figure 4c). In the MIRI-enalapril group (Figure 4d), a relatively restored tubular and glomerular structure was noted. In the MIRI-acarbose-treated group (Figure 4e), pronounced regenerative changes in the tubular and glomerular apparatus were seen. A fully restored tubular and glomerular microarchitecture was observed in the diabetic group treated with ETBhd (Figure 4f).

## 4. Discussion

Metabolically induced renal injury (MIRI) or diabetic nephropathy (DN) is a serious complication of diabetes mellitus. A large number of patients with diabetes may develop chronic kidney disease that progresses to end-stage renal failure. Although the exact pathogenesis of MIRI is not yet well established, several factors, such as hyperglycemia, hyperlipidemia, hypertension, and proteinuria, contribute to the progression of renal impairment in diabetic nephropathy. MIRI is confirmed by measuring blood urea and creatinine concentrations, creatinine clearance, and proteinuria. The control of hyperglycemia and blood pressure has been shown to reduce proteinuria, which is the main sign of glomerular damage in MIRI [3].

The pathogenesis of MIRI is associated with hyperglycemia, activating various metabolic, hemodynamic, inflammatory, and fibrotic mechanisms and pathways. A growing number of studies have reported that a variety of growth factors are involved in these signaling pathways [29]. Various mediators, such as inflammatory cytokines, reactive oxygen species, and transforming growth factor β, are shared between these pathways, leading to significant overlap and interactions between them. Cytokines such as TGF-β, ICAM-1, VCAM-1, TNF-α, CTGF, VEGF, and plasminogen activator inhibitor (PAI)-1 are released and are involved in various diabetic complications. VEGF, TGF-β1, and FGF-23 have been shown to significantly contribute to DKD [17,30,31,32]. Studies have shown that high levels of FGF23 induce inflammation in patients with DN [33]. A study performed by Zanchi et al. in 2013 showed that angiotensin-converting enzyme (ACE) inhibitors protect against kidney damage in patients with DN not only by reducing blood pressure but also by modulating plasma levels of FGF23 and its secretion in the kidney [34].

In the present study, we focused on the antidiabetic and nephroprotective properties of ETB and sought to determine whether the plant extract administration was beneficial for attenuating hyperglycemia, hypertension, proteinuria, and oxidative stress markers and enzymes in rats. In these experiments, 8 weeks of treatment with ETB at 150 and 300 mg/kg/p.o. improved markers of oxidative stress MDA and GSH; the activity of the antioxidant enzymes GPx, GR, GST, and SOD; and tended to reduce blood glucose levels and systolic blood pressure.

Through 8 weeks of consumption of a high-fat diet, the intake of a 10% fructose solution and the intraperitoneal administration of streptozotocin induced type 2 diabetes with some of its associated complications such as increased blood pressure, oxidative stress, and increased markers characterizing renal dysfunction, described in the results. Therefore, in this study, metabolic syndrome is also associated with metabolically induced renal damage. In this sense, we put an equal sign between animals with induced metabolic syndrome (METS) and metabolically induced renal injury (MIRI). MIRI was confirmed by histopathological findings of increased blood pressure and levels of serum urea and creatinine, ketonuria, glycosuria, and proteinuria.

The pathogenesis of diabetic nephropathy is associated with hyperglycemia, which activates various metabolic and hemodynamic pathways and oxidative stress. These processes activate the RAAS system and lead to an increase in blood pressure. In diabetic nephropathy, oxidative stress activates the renin-angiotensin-aldosterone system (RAAS) that damages the kidney [17]. Hyperglycemia causes glomerular hyperfiltration and intraglomerular hypertension by activating the local RAAS. The high blood pressure in the glomerulus accelerates renal vascular complications.

In the context of this pathophysiology, the use of drugs from the ACE inhibitor group is of particular importance, so we applied enalapril. It is widely used in clinical practice, not only to reduce high blood pressure, but also to slow the progression of kidney damage in patients with diabetic nephropathy. We applied enalapril as a positive control for antihypertensive action, since in the course of the experiment, after inducing diabetes, in addition to high blood sugar, we also found high blood pressure in animals. So, as a control drug for hypertension, we chose the ACE inhibitor most commonly used in practice.

For the control of hyperglycemia, acarbose was administered because in our previous experiments, we established good alpha-glucosidase inhibitory potential of *Tanacetum balsamita* [9], and an extract of this species was used in another in vivo diabetes study [6].

Oxidative stress plays an important role in the progression of MIRI. Diabetes complications were caused by prolonged exposure to high levels of glucose, leading to mitochondrial overproduction of reactive oxygen species (ROS). They cause renal damage characterized by structural and functional changes in glomerular and renal tubular cells. Normally, renal cells defend themselves against ROS damage with various antioxidants, including the antioxidant enzymes superoxide dismutase, catalase, and glutathione peroxidase. Among these antioxidant enzymes, SOD is the most potent. Transgenic overexpression of SOD in diabetic mice has been shown to reduce oxidative stress, albuminuria, and imbalance in the glomerular matrix. Several studies have shown that increased lipid metabolism and ROS may be associated with the activation of renal tissue damage. SOD and catalase are known to be regulated by the nuclear transcription factor, Nrf2, which is a key to the inhibition of oxidative stress and lipid accumulation in type 2 DN and high-fat diets [35].

In diabetic nephropathy, oxidative stress activates the renin-angiotensin-aldosterone system (RAAS) that damages the kidney [17]. Hyperglycemia causes glomerular hyperfiltration and intraglomerular hypertension by activating the local RAAS. The high blood pressure in the glomerulus accelerates renal vascular complications. Moreover, angiotensin II produced by the RAAS can cause podocyte injury by increasing the production of reactive oxygen species (ROS) [36]. ACE inhibitors combined with antioxidants and antihyperglycemic drugs in MIRI are promising therapeutic modality against this complex pathology. Because of this, we used enalapril as a positive control in this study.

The obtained results are consistent with previous data, demonstrating that enalapril treatment significantly enhanced the renal total antioxidant status and SOD activity, reduced MDA levels, and prevented the renal dysfunction and histopathological changes in hypertensive animals [37]. Our results indicated that, besides its hypotensive and renoprotective effects, enalapril treatment also diminished oxidative stress in the kidneys of hypertensive animals. In similar studies, the deteriorated kidney lipid peroxidation, GSH content and GST, and catalase activities in diabetic rats were significantly ameliorated as a result of treatment with enalapril. It was concluded that this ACE inhibitor has an antioxidant effect and can prevent STZ diabetes-induced nephropathy through amelioration of the glycemic state and antioxidant defense system together with the suppression of oxidative stress, inflammation, and apoptosis [38].

The ETB at a high dose has notable impact on the markers of oxidative stress and the activity of the antioxidant enzymes (Table 1 and Figure 2). We demonstrate that ETB can prevent MIRI-induced elevation of MDA and decreases of GSH, GPx, GR, GST, and SOD, which hold significance for reducing oxidative stress associated with MIRI. Taking together our results on the phytochemistry and pharmacological activity of ETB, it could be hypothesized that the 3,5-dicaffeoylquinic (DCQA), chlorogenic, and rosmarinic acids in the extract are the main antioxidant determinants.

ETB phytochemical profiling has been published elsewhere [8]. From a variety of mono-, di-, and triacylquinic acids including caffeoyl-, coumaroyl-, and feruloylqunic acids, dicaffeoylquinic, caffeoyl-coumaroylquinic, caffeoyl-feruloylquinic, and tricaffeoylquinic acids delineated the profile of specialized metabolites of ETB. It was dominated by 3,5-dicaffeoylquinic acid (3,5diCQA) in UHPLC-DAD analysis, followed by chlorogenic acid (CGA) and rosmarinic acid.

Consistent with previously published data on diCQA-rich plant extract, a significant decrease in blood glucose levels and a mildly antihypertensive effect were achieved in diabetic animals [4].

Antioxidant and organ protective effects of these plant-derived caffeic acid conjugates are broadly discussed in the scientific literature. Caffeoylquinic acids are some of the chemophenetically significant specialized metabolites found in plants of the family Asteraceae Dumort., possessing a broad spectrum of biological activities [39].

The chlorogenic acid derivative 3,5-diCQA with multiple phenolic hydroxyl groups can neutralize free radicals, thereby alleviating oxidative stress-induced cellular damage [40]. In the study performed by Acikara et al. [41] 3,5-O-dicaffeoylquinic acid exhibited the most potent antioxidant activity among the compounds studied. Yin et al. demonstrated that the treatment of diabetic mice with dicaffeoylquinic acids-enriched extract of *Gynura divaricata* increased glutathione peroxidase and superoxide dismutase activities while mitigating malondialdehyde levels [42]. El-Askary et al. revealed the potential mechanisms involved in the protective effect of dicaffeoylquinic acids from *Artemisia annua* leaves against diabetes and its complications. According to their study the dicaffeoylquinic acid derivatives were capable of inhibiting the activities of the DPP IV, α-amylase, and α-glucosidase enzymes in vitro. Moreover, these compounds exhibited potential effectiveness in preventing diabetes complications via inhibiting aldose reductase enzymes and alleviating oxidative stress [4].

In our previous studies, we found antihypertensive, antidiabetic, antioxidant, hepatoprotective and nephroprotective effects of 3,5-DCQA isolated from *Geigeria alata* on diabetic hypertensive animals [38].

The investigation by Ahmed OM et al. revealed that the treatment of diabetic Wistar rats with enalapril led to significant decreases in the elevated serum urea, uric acid, creatinine, sodium, and potassium levels; thereby reflecting the improvement of the impaired kidney function [38]. Moreover, the treatment of diabetic rats with enalapril successfully prevented the diabetes-induced histopathological deleterious changes of the kidney that was also confirmed in the present investigation.

Acarbose did not change these parameters significantly. The low dose of the extract reduced the levels of both markers by about 22%, and the high dose statistically significantly reduced the concentrations of urea and creatinine in serum by 35%, respectively, compared to that in diabetic rats (Figure 3a,b).

Bao et al. investigated the renoprotective effect of chlorogenic acid (CGA) in vivo in a high-fat diet (HFD)/streptozotocin (STZ)-induced diabetic rat kidney [43]. The results of their study show that the levels of serum creatinine, blood urea, and urinary protein excretion in diabetic rats were significantly decreased after CGA intervention. We received similar results after ETB administration to rats with MIRI. As mentioned above, *T. balsamita* extract is rich in CGA. CGA administration can activate the Nrf2 pathway and inhibit NLRP3 inflammasome activation. Notably, Nrf2 siRNA transfection nullified the inhibitory effects of CGA on NLRP3 inflammasome activation in vitro. To summarize, Bao et al. provided evidence that chlorogenic acid can slow the progression of diabetic nephropathy [43]. The authors concluded: (1) CGA mitigates diabetic renal oxidative stress via regulating the Nrf2 pathway; (2) CGA relieves diabetic renal inflammation by inhibiting NF-ĸB pathway; (3) CGA-induced activation of Nrf2/HO-1 interacts with the inhibition of NF-ĸB, as each begets and amplifies the other; (4) CGA could be a potential therapeutic agent in the treatment of diabetic nephropathy [43].

In our investigation as well as in the studies of Poloni and Rotta, it was found that diabetes and MIRI were associated with ketonuria, proteinuria, glycosuria, etc. (Table 2) [44,45]. Enalapril and ETB significantly ameliorated the quantity of these end-products in the urine of treated animals, as was also displayed in the works of Akhtar et al. [46] and Fauzi et al. [47], who provided evidence about the nephroprotective potential of some plant species rich in CGA.

Control of hyperglycemia and blood pressure has been shown to reduce proteinuria, which is the main sign of glomerular lesions in MIRI. As mentioned above, ETB effectively decreased blood glucose levels and the higher blood pressure induced by HFD and STZ administration in rats.

The study by Bao et al. [43] showed that diabetic rats had notable glomerular hypertrophy, mesangial matrix deposition, renal tubular vacuolar degeneration, and renal tubular dilatation. The results demonstrated that CGA significantly affected renal morphological changes in rats with induced DN, and the authors concluded that their study provided evidence that CGA can slow the progression of MIRI, and the effect is not only associated with antioxidant effects but also with suppression of NLRP3 inflammasome activation, suggesting its therapeutic implications for MIRI [43].

It shows multidimensional functions, including neuroprotection for diabetic peripheral neuropathy, anti-inflammation, anti-oxidation, mitigation of diabetes mellitus, and liver and kidney injuries. Mechanistically, it acts through the modulation of anti-inflammation/oxidation and metabolic homeostasis [48]. The pharmacological properties of DCQAs have been studied over the last few decades, suggesting antioxidative, anti-inflammatory, antimicrobial, hypoglycaemic, cardiovascular protective, neuroprotective, and hepatoprotective effects [49].

Flavonoids are the other class of secondary metabolites widely spread in Asteraceae taxa. Our results are in accordance with the previous funding for the protective effects of flavonoids against chemically induced renal toxicity. Flavonoids mitigated arterial hypertension, oxidative stress, inflammatory diseases, and changes in vascular health and have shown promising results in treating acute and chronic nephropathies of renal fibrosis [50]. Flavonoids may also act directly on the renal parenchyma and interfere with signaling pathways, affecting the development of renal injury and exerting nephroprotective effects in diabetic nephropathy, glomerulonephritis, and chemically induced kidney insufficiency [51]. Flavonoids exert protective effects by decreasing the excessive ROS level or activating renal enzymatic and non-enzymatic antioxidants via different pathways, including modulating the Nrf2-antioxidant pathway [50].

Recently, Hu et al. found that flavonoids play a therapeutic role in diabetic nephropathy mainly by regulating oxidative stress and inflammation. Quercetin, apigenin, and luteolin were found to be capable of alleviating oxidative stress related to the Nrf-2/GSH, ROS production, HO-1, TGF-β1, and AGEs/RAGE. Regarding inflammatory responses, they are thought to be essential. Quercetin, kaempferol, myricetin, and rutin were confirmed to influence the IL-1, IL-6β, TNF-α, SIRT1, NF-κB, and TGF-β1/smad targets. As a result, flavonoids promote podocyte autophagy and inhibit the overactivity of the RAAS by suppressing the upstream oxidative stress and inflammatory pathways, ultimately alleviating DN. The above results indicate that flavonoids are promising drugs for the treatment of diabetic nephropathy [52].

Isorhamnetin improves fasting blood glucose, renal, and lipid profiles with increased autophagosomes in renal tissues. It suppresses miRNA regulation of autophagy genes [53]. Isorhamnetin was indicated for the prevention and treatment of diabetes and its complications. It activates the JAK2/STAT pathway and promotes glucose uptake by increasing GLUT4 translocation in different signaling pathways in skeletal muscle cells at a low concentration range, thereby providing beneficial functions for preventing hyperglycemia and maintaining glucose homeostasis [54].

Although current treatment options for DN have shown progress over previous strategies, their efficacy remains limited due to the residual risk of disease progression. Detailed elucidation of the molecular mechanisms inducing DN progression is imperative in order to develop new strategies and innovative therapies for this condition [31].

## 5. Conclusions

Our results suggest that ETB possesses antidiabetic and nephroprotective potential on MIRI through mitigating the hyperglycemia, hypertension, oxidative stress, and serum biochemistry characterizing kidney function. All these parameters reflect the renal histological microarchitecture. ETB benefits on MIRI could be related to the high levels of mono- and dicaffeoylquinic acids together with a series of methoxylated flavones and flavonols, which may hold significance for its antidiabetic and nephroprotective activity. ETB has prospects as a food supplement for relieving the symptoms of MIRI.

## Figures and Tables

**Table 1:** Activity of antioxidant enzymes in kidney homogenates in all experimental groups.

Enzyme Activity (nmol/min/mg)	Controls	ETBld	ETBhd	MIRI	MIRI + enlp	MIRI + acarb	MIRI + ETBld	MIRI + ETBhd
GPx	27.35 ± 1.08	26.5 ± 1.29	28.37 ± 0.47	18.75 ± 1.71 *	25.27 ± 0.57 ^+^	22.02 ± 1.48	24.37 ± 1.88 ^+^	27.45 ± 2.51 ^+^
GR	52.42 ± 0.61	50.7 ± 2.58	52.95 ± 2.16	41.27 ± 2.39 *	47.72 ± 1.69 ^+^	44.7 ± 3.18	48.2 ± 2.17 ^+^	50.2 ± 0.91 ^+^
GST	13.17 ± 0.67	13.10 ± 0.62	12.32 ± 0.68	8.90 ± 0.68 *	12.27 ± 0.63 ^+^	10.42 ± 1.22	11.0 ± 0.81 ^+^	11.77 ± 0.68 ^+^
SOD	0.32 ± 0.015	0.36 ± 0.021	0.35 ± 0.023	0.23 ± 0.017	0.41± 0.026 *	0.38 ± 0.012	0.44 ± 0.022	0.45± 0.031

* *p* < 0.05 vs. control; ^+^ *p* < 0.05 vs. MIRI; treatment: as described in the experimental design section.

**Table 2:** Urine parameters, measured with test strips.

Urine Parameters	Controls	ETBld	ETBhd	MIRI	MIRI + enlp	MIRI + acarb	MIRI + ETBld	MIRI + ETBhd
Ketone mmol/L	0.0 ± 0	0.0 ± 0	0.0 ± 0	12.0 ± 4.6 *	3.5 ± 3.3	4.38 ± 2.7	0.88 ± 0.7 ^+^	0.63 ± 0.6 ^+^
Protein g/L	0.0 ± 0	0.0 ± 0	0.0 ± 0	13.25 ± 7.2 *	2.5 ± 1.0 ^+^	2.0 ± 1.1 ^+^	0.65 ± 0.4 ^+^	0.58 ± 0.5 ^+^
Glucose mmol/L	0.0 ± 0	0.0 ± 0	0.0 ± 0	41.25 ± 14 *	10.0 ± 5.7 ^+^	8.75 ± 2.5 ^+^	7.5 ± 2.8 ^+^	2.5 ± 2.8 ^+^
pH	7.25 ± 0.2	7.13 ± 0.2	7.0 ± 0.5	5.25 ± 0.5 *	5.75 ± 0.5	7 ± 0.5 ^+^	7.25 ± 0.2 ^+^	7.5 ± 0.5 ^+^
Volume ml/24 h	32.5 ± 4.2	38.6 ± 3.5	42.3 ± 3.8	66.2 ± 5.2 *	56.2 ± 6.4	48.6 ± 3.8 ^+^	47.2 ± 3.2 ^+^	46.5 ± 4.1 ^+^

* *p* < 0.05 vs. control; ^+^ *p* < 0.05 vs. MIRI; treatment: as described in the experimental design section.

## Data Availability

The original contributions presented in the study are included in the article.

## Supplementary Materials

The following supporting information can be downloaded at: https://www.mdpi.com/article/10.3390/cimb47040293/s1.

## Abbreviations

The following abbreviations are used in this manuscript:

MIRI	Metabolic-induced renal injury
MAFLD	Metabolic-associated fatty liver disease
ETB	Tanacetum balsamita leaves extract
DCQA	Dicaffeoylquinic acids
ESRD	End-stage renal disease
DN	Diabetic nephropathy
MDA	Malondialdehyde
GR	Glutathione reductase
GPx	Glutathione peroxidase
GST	Glutathione-S-transferase
SOD	Superoxide dismutase
RAS	Renin-angiotensin system
GLP-1	Glucagon-like peptide-1
nsMRAs	Nonsteroidal mineralocorticoid receptor antagonists
(UHPLC-DAD)	Ultra-high-performance liquid chromatography–Diode Array Detection
GAE	Gallic acid equivalent
QE	Quercetin equivalent
CGA	Chlorogenic acid
DE	Dry extract
ROS	Reactive oxygen species
JAK/STAT	Janus Kinase/Signal Transducer and Activator of Transcription
Nrf-2	Nuclear factor erythroid 2-related factor 2
HFD	High-fat diet
GLUT4	Glucose transporter 4
TGF	Transforming growth factor
TNF	Tumor necrosis factor
