# Supplementary material for: Nephroprotective Effects of Tanacetum balsamita Extract on Metabolic-Induced Renal Injury (MIRI) in Rats
Source: Curr Issues Mol Biol. 2025 Apr 21;47(4):293. doi: 10.3390/cimb47040293 (PMC12026245; doi:10.3390/cimb47040293)
Supplement: Supplementary file 1 [file cimb-47-00293-s001.zip › cimb-3568998-supplementary.pdf]

Nephroprotective effects of *Tanacetum balsamita* extract on Metabolic-Induced Renal Injury (MIRI) in rats

Rumyana Simeonova [1,*], Reneta Gevrenova [2], Lyubomir Marinov [1], Yonko Savov [3], and Dimitrina Zheleva-Dimitrova [2,*]

[1] Department of Pharmacology, Pharmacotherapy and Toxicology, Faculty of Pharmacy, Medical University of Sofia, 1000 Sofia, Bulgaria; lmarinov@pharmfac.mu-sofia.bg
[2] Department of Pharmacognosy, Faculty of Pharmacy, Medical University of Sofia, 1000 Sofia, Bulgaria; rgevrenova@pharmfac.mu-sofia.bg
[3] Institute of Emergency Medicine "N. I. Pirogov", Bul. Totleben 21, 1000 Sofia, Bulgaria; yonko_savov@hotmail.com
* Correspondence: rsimeonova@pharmfac.mu-sofia.bg (R.S.); dzheleva@pharmfac.mu-sofia.bg (D.Z.-D.)

Supplemental material

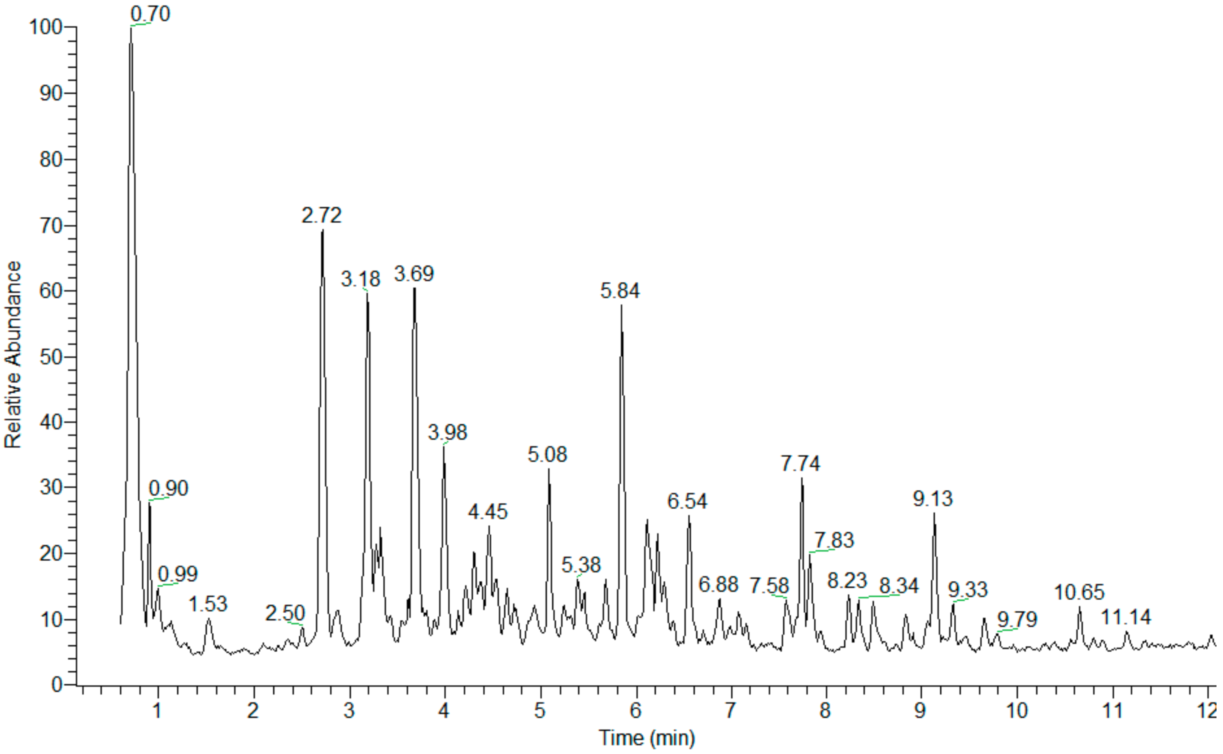

Figure S1. Total ion chromatogram in negative ion mode of *T. balsamita* leaf extract (ETB).

Table S1. Secondary metabolites in ETB assayed by UHPLC-HRMS [1].

No	Identified/Tentatively Annotated Compound	Molecular Formula	Exact Mass [M-H]$^-$	t_R (min)	Δ ppm
	**Hydroxybenzoic, Hydroxycinnamic and Acylquinic Acids, and Derivatives**				
**1**	protocatechuic acid-*O*-hexoside	$C_{13}H_{16}O_9$	315.0727	1.72	0.840
**2**	hydroxybenzoic acid-pentosylhexoside	$C_{18}H_{23}O_{12}$	431.1198	1.83	0.582
**3**	protocatechuic acid [a]	$C_7H_6O_4$	153.0182	2.05	−8.574
**4**	protocatechuic acid-*O*-hexoside isomer	$C_{13}H_{16}O_9$	315.0753	2.15	0.840
**5**	*p*-hydroxyphenylacetic acid 1-*O*-hexoside	$C_{14}H_{18}O_8$	313.0727	2.17	4.022
**6**	syringic acid [a]	$C_9H_{10}O_5$	197.0455	2.29	−5.819
**7**	syringic acid 4-*O*-hexoside	$C_{15}H_{20}O_{10}$	359.0984	2.30	0.362
**8**	neochlorogenic (3-caffeoylquinic) acid [a]	$C_{16}H_{18}O_9$	353.0867	2.38	0.240
**9**	caffeic acid- *O*-hexoside	$C_{15}H_{18}O_9$	341.0867	2.42	−3.153
**10**	vanillyl-*O*-hexose	$C_{14}H_{18}O_9$	329.0875	2.50	−1.049
**11**	gentisic acid-O-hexoside	$C_{13}H_{16}O_9$	315.0727	2.58	0.555
**12**	aesculetin-*O*-hexoside	$C_{15}H_{15}O_9$	339.0724	2.69	0.781
**13**	vanillic acid [a]	$C_8H_8O_4$	167.0350	3.03	−7.735
**14**	caffeoylgluconic acid	$C_{15}H_{18}O_{10}$	357.0827	2.82	2.185
**15**	*O*-caffeoyl hexose	$C_{15}H_{18}O_9$	341.0867	2.83	−1.012
**16**	4-hydroxybenzoic acid [a]	$C_7H_6O_3$	137.0230	2.86	−10.052
**17**	*p*-hydroxyphenylacetic acid-*O*-hexoside isomer	$C_{14}H_{18}O_8$	313.0936	3.01	1.754
**18**	hydroxybenzoic acid-*O*-hexoside	$C_{13}H_{16}O_8$	299.0778	3.02	2.238
**19**	dihydroxyphenylacetic acid-*O*-pentosylhexoside	$C_{22}H_{21}O_{11}$	461.1115	3.03	5.520
**20**	caffeoylgluconic acid isomer	$C_{15}H_{18}O_{10}$	357.0827	3.07	0.812
**21**	caffeic acid-*O*-hexoside isomer	$C_{15}H_{18}O_9$	341.0867	3.12	0.160
**22**	quinic acid	$C_7H_{12}O_6$	191.0561	3.19	−5.921
**23**	chlorogenic (5-caffeoylquinic) acid [a]	$C_{16}H_{18}O_9$	353.0867	3.19	0.495
**24**	caffeic acid-*O*-hexoside	$C_{15}H_{18}O_9$	341.0867	3.27	−0.104
**25**	coumaric acid-*O*-hexoside	$C_{15}H_{18}O_8$	325.0930	3.32	0.305
**26**	*p*-hydroxyphenylacetic acid-*O*-hexoside isomer	$C_{14}H_{18}O_8$	313.0934	3.31	0.988
**27**	*p*-coumaric acid [a]	$C_9H_8O_3$	163.0389	3.35	−8.510
**28**	4-caffeoylquinic acid [a]	$C_{16}H_{18}O_9$	353.0867	3.36	0.551
**29**	caffeoylgluconic acid isomer	$C_{15}H_{18}O_{10}$	357.0827	3.41	−2.100
**30**	3-feruloylquinic acid	$C_{17}H_{20}O_9$	367.1035	3.43	−1.921
**31**	*p*-hydroxyphenylacetic acid [a]	$C_8H_8O_3$	151.0401	3.47	−9.715
**32**	caffeic acid [a]	$C_9H_8O_4$	179.0338	3.54	−6.211
**33**	gentisic acid [a]	$C_7H_6O_4$	153.0182	3.84	−8.901
**34**	5-*p*-coumaroylquinic acid	$C_{16}H_{18}O_8$	337.0929	3.96	1.096
**35**	3-hydroxy-dihydrocaffeoyl-5-caffeoylquinic acid	$C_{25}H_{26}O_{13}$	533.1301	4.05	−0.570
**36**	5-feruloylquinic acid	$C_{17}H_{20}O_9$	367.1035	4.41	−0.015
**37**	dihydroxyphenylacetic acid	$C_8H_8O_4$	167.0341	4.41	−5.520
**38**	1-caffeoyl-3-hydroxy-dihydrocaffeoylquinic acid	$C_{25}H_{26}O_{13}$	533.1301	4.45	2.412
**39**	coumaric acid-*O*-hexoside isomer	$C_{15}H_{18}O_8$	325.0931	4.45	0.398
**40**	*m*-coumaric acid [a]	$C_9H_8O_3$	163.0389	4.46	−7.651
**41**	*o*-coumaric acid [a]	$C_9H_8O_3$	163.0389	4.56	−8.142
**42**	5-*p*-coumaroylquinic acid isomer	$C_{16}H_{18}O_8$	337.0929	4.62	0.829
**43**	4-feruloylquinic acid	$C_{17}H_{20}O_9$	367.1035	4.68	0.122
**44**	3,5-dicaffeoylquinic acid-hexoside	$C_{31}H_{34}O_{17}$	677.1512	5.16	3.850
**45**	4,5-dicaffeoylquinic acid-hexoside	$C_{31}H_{34}O_{17}$	677.1512	5.56	0.779
**46**	3,4-dicaffeoylquinic acid [a]	$C_{25}H_{24}O_{12}$	515.1195	5.70	0.487
**47**	3-dehydrocaffeoyl-5-caffeoylquinic acid	$C_{25}H_{22}O_{12}$	513.1038	5.85	0.898
**48**	3,5-dicaffeoylquinic acid [a]	$C_{25}H_{24}O_{12}$	515.1195	5.87	0.137

**49**	dihydroxyphenylacetic acid-*O*-dipentosyl-hexoside	$C_{27}H_{29}O_{15}$	593.1543	6.19	5.238
**50**	4,5-dicaffeoylquinic acid	$C_{25}H_{24}O_{12}$	515.1195	6.23	0.390
**51**	shikimic acid	$C_{7}H_{10}O_{5}$	173.0455	6.22	−7.147
**52**	rosmarinic acid [a]	$C_{18}H_{16}O_{8}$	359.0778	6.33	1.781
**53**	3-feruloyl-4-caffeoylquinic acid	$C_{26}H_{26}O_{12}$	529.1351	6.50	0.096
**54**	3-*p*-coumaroyl-5-caffeoylquinic acid	$C_{25}H_{24}O_{11}$	499.1246	6.52	1.694
**55**	1-*p*-coumaroyl-5-caffeoylquinic acid	$C_{25}H_{24}O_{11}$	499.1246	6.80	−2.173
**56**	3-feruloyl-5-caffeoylquinic acid	$C_{26}H_{26}O_{12}$	529.1351	6.82	0.190
**57**	4-feruloyl-5-caffeoyl quinic acid	$C_{26}H_{26}O_{12}$	529.1351	7.02	0.549
**58**	4-caffeoyl-5-feruloylquinic acid	$C_{26}H_{26}O_{12}$	529.1351	7.18	1.343
**59**	4-caffeoyl-5-*p*-coumaroylquinic acid	$C_{25}H_{24}O_{11}$	499.1246	7.63	1.453
60	3,4,5-tricaffeoylquinic acid	$C_{34}H_{30}O_{15}$	677.1512	7.78	0.748
	**Flavonoids**				
**61**	naringenin 6, 8 di*C*-hexoside	$C_{27}H_{32}O_{15}$	595.1678	3.64	1.994
**62**	apigenin 6, 8-di*C*-hexoside	$C_{27}H_{29}O_{15}$	593.1512	4.04	0.905
**63**	homoorientin (luteolin 6-*C*-glucoside) [a]	$C_{21}H_{20}O_{11}$	447.0933	4.54	0.225
**64**	luteolin *O*-hexuronosyl-*O*-hexoside	$C_{27}H_{28}O_{17}$	623.1264	4.72	1.457
**65**	rutin [a]	$C_{27}H_{30}O_{16}$	609.1464	5.08	0.972
**66**	luteolin *O*-pentosylhexoside	$C_{26}H_{28}O_{15}$	579.1360	5.09	1.394
**67**	isoquercitrin [a]	$C_{21}H_{20}O_{12}$	463.0886	5.18	1.103
**68**	hyperoside [a]	$C_{21}H_{20}O_{12}$	463.0887	5.29	1.218
**69**	nepetin *O*-pentosylhexoside	$C_{27}H_{30}O_{16}$	609.1468	5.35	−5.123
**70**	luteolin 7-*O*-rutinoside	$C_{27}H_{30}O_{15}$	593.1512	5.22	1.006
**71**	luteolin 7-*O*-glucoside [a]	$C_{21}H_{20}O_{11}$	447.0933	5.31	0.437
**72**	luteolin *O*-hexuronide [b]	$C_{21}H_{18}O_{12}$	461.0736	5.38	0.978
**73**	isorhamnetin *O*-hexuronide	$C_{22}H_{20}O_{13}$	491.0832	5.47	0.970
**74**	kaempferol 7-*O*-rutinoside	$C_{27}H_{30}O_{15}$	593.1520	5.65	1.124
**75**	nepetin *O*-hexoside	$C_{22}H_{22}O_{12}$	477.1038	5.67	−0.253
**76**	axillarin *O*- pentosylhexoside	$C_{28}H_{32}O_{17}$	639.1567	5.74	0.012
**77**	apigenin *O*-pentosylhexoside	$C_{26}H_{28}O_{14}$	563.1406	5.75	0.961
**78**	apigenin 7-*O*-rutinoside	$C_{27}H_{30}O_{14}$	577.1570	5.82	1.250
**79**	isorhamnetin 3-*O*-glucoside [a]	$C_{22}H_{22}O_{12}$	477.1042	5.90	0.253
**80**	hispidulin *O*-pentosylhexoside	$C_{27}H_{30}O_{15}$	593.1512	5.93	1.832
**81**	isorhamnetin *O*-pentoside	$C_{21}H_{19}O_{11}$	447.0935	6.02	0.437
**82**	chrysoeriol *O*-pentosylhexoside	$C_{27}H_{30}O_{15}$	593.1512	6.04	2.962
**83**	apigenin *O*-hexuronide	$C_{21}H_{18}O_{11}$	445.0787	6.13	0.484
**84**	kaempferol 3-*O*-glucoside [a]	$C_{21}H_{19}O_{11}$	447.0935	6.21	0.504
**85**	jaceosidin *O*-hexuronide	$C_{23}H_{22}O_{13}$	505.0988	6.33	−2.731
**86**	chrysoeriol *O*-hexuronide	$C_{22}H_{20}O_{12}$	475.0882	6.34	0.254
**87**	jaceosidin *O*-hexoside	$C_{23}H_{24}O_{12}$	491.1195	6.50	0.877
88	eupatilin *O*-hexoside	$C_{24}H_{26}O_{12}$	505.1351	7.49	0.932
**89**	luteolin [a]	$C_{15}H_{10}O_{6}$	285.0405	7.58	−0.636
**90**	quercetin [a]	$C_{15}H_{10}O_{7}$	301.0354	7.63	−0.019
**91**	patuletin (6-methoxyquercetin)	$C_{16}H_{12}O_{8}$	331.0464	7.72	0.149
**92**	nepetin (6-methoxyluteolin)	$C_{16}H_{12}O_{7}$	315.0514	7.75	1.251
**93**	spinacetin	$C_{17}H_{14}O_{8}$	345.0616	7.85	−0.726
**94**	axillarin	$C_{17}H_{14}O_{8}$	345.0616	8.25	−0.205
**95**	apigenin [a]	$C_{15}H_{10}O_{5}$	269.0457	8.62	−1.942
**96**	hispidulin (scutellarein-6-methyl ether) [a]	$C_{16}H_{12}O_{6}$	299.0563	8.84	−0.372
**97**	quercetagetin-3,6,3′(4′)-trimethyl ether	$C_{18}H_{16}O_{8}$	359.0772	9.08	1.112
**98**	isorhamnetin[a]	$C_{16}H_{12}O_{7}$	315.0512	9.11	−0.551
**99**	jaceosidin (6-hydroxyluteolin-6,3′-dimethyl ether) [a]	$C_{17}H_{14}O_{7}$	329.0677	9.15	0.073
**100**	cirsiliol	$C_{17}H_{14}O_{7}$	329.0677	9.47	0.954

**101**	quercetagetin-3,6,3′(4′)-trimethyl ether	$C_{18}H_{16}O_8$	359.0772	9.66	1.196
**102**	cirsimaritin (6-hydroxyapigenin-6,7-dimethyl ether) [a]	$C_{17}H_{14}O_6$	313.0719	10.39	0.059
**103**	eupatilin/santin	$C_{18}H_{16}O_7$	343.0812	10.68	−0.047

[a] Compared to a reference standard; [b] reported for the first time; * annotation was done in (+) ESI-MS/MS

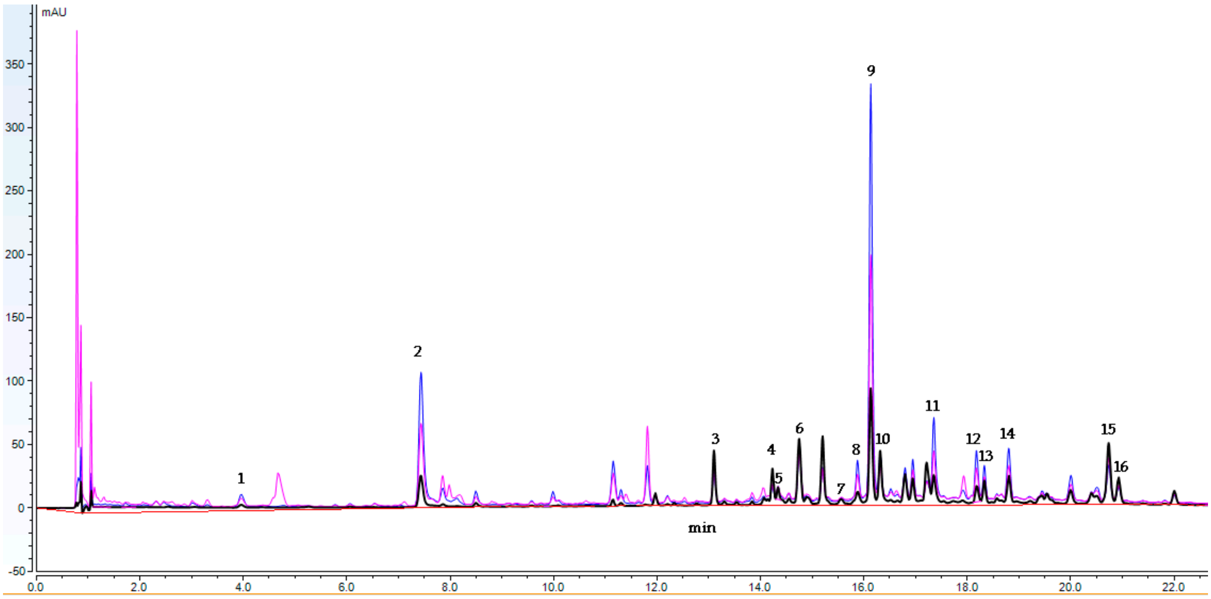

Figure S2. UHPLC-DAD chromatograms of ETB; wavelengths: 360 nm, 310 nm, 280 nm [2].

Table S2. Content (µg/mg dry extract) of compounds assayed in ETB using UHPLC-DAD [2].

№	Analyte	t_R	Content (µg/mg de)
1.	neochlorogenic acid	4.38	2.817±0.297
2.	chlorogenic acid	7.70	17.967±0.113
3.	orientin	13.10	4.603±0.401
4.	hyperoside	14.23	3.922±0.356
5.	rutin	14.34	0.483±0.017
6.	luteolin 7-*O*-glucoside	14.75	3.671±0.316
7.	kaempferol 3-*O*-rutinoside	15.59	0.857±0.015
8.	3,4-diCQA	15.91	7.252±0.921
9.	3,5-diCQA	16.17	69.129±9.344
10	isorhamnetin 3-*O*-glucoside	16.34	5.720±0.584
11	rosmarinic acid	17.38	12.950±2.627
12	AQA1	18.20	9.491±0.250
13	AQA2	18.35	6.586±0.072
14	AQA3	18.82	8.130±0.505
15	luteolin	20.76	4.049±0.393
16	nepetin	20.94	1.039±0.141

References

1. Gevrenova, R.; Zengin, G.; Sinan, K.I.; Zheleva-Dimitrova, D.; Balabanova, V.; Kolmayer, M.; Voynikov, Y.; Joubert, O. An In-Depth Study of Metabolite Profile and Biological Potential of Tanacetum Balsamita L. (Costmary). *Plants* **2022**, *12*, 22, doi:10.3390/plants12010022.

2. Mihaylova, R.; Gevrenova, R.; Petrova, A.; Savov, Y.; Zheleva-Dimitrova, D.; Balabanova, V.; Momekov, G.; Simeonova, R. Mitigating Effects of Tanacetum Balsamita L. on Metabolic Dysfunction-Associated Fatty Liver Disease (MAFLD). *Plants* **2024**, *13*, 2086, doi:10.3390/plants13152086.
